# Altruistic donation to improve survey responses: a global randomized trial

**DOI:** 10.1186/s13104-019-4146-y

**Published:** 2019-02-28

**Authors:** Andrew J. Cohen, Sam Washington, Christi Butler, Puneet Kamal, German Patino, Anas Tresh, Jorge Mena, Medina Ndoye, Benjamin N. Breyer

**Affiliations:** 1 Department of Urology, Zuckerberg San Francisco General Hospital and Trauma Center, University of California, San Francisco, 1001 Potrero Suite 3A, San Francisco, CA 94110 USA; 2 Department of Biostatistics and Epidemiology, University of California-San Francisco, San Francisco, CA USA

**Keywords:** Incentives, Survey response, Global, Donation, Nested randomized control

## Abstract

**Objective:**

Web-based platforms have revolutionized the ability for researchers to perform global survey research. Methods to incentivize participation have been singularly focused on European and North American participants with varied results. With an ever increasing proportion of biomedical research being performed in non-western countries, assessment of novel methods to improve global survey response is timely and necessary. To that end, we created a three-arm nested randomized control trial (RCT) within a prospective cohort study to assess the impact of incentives on survey responsiveness in a global audience of biomedical researchers.

**Results:**

Email invitations were sent to authors and editors involved in online publishing totaling 2426 participants from 111 countries. Overall we observed a 13.0% response rate: 13.3% for the control group, 14.4% for a group entered to win a gift card, and 11.1% for a group whose participation lead to donation to charity (p = 0.17). Year of publication nor country impacted response rate. Within subgroups, editors were significantly less likely to respond to the survey as compared to authors (6.5% vs. 18.9%; p-value < 0.01). With power to detect a 4.8% difference among groups, we could not detect an impact of incentives on global survey response.

## Introduction

Achieving adequate survey response has become increasingly challenging as surveys compete for subjects’ time and attention [1]. While post-cards and phone reminders have been a staple of survey-based research for decades, [2, 3] the integration of web-based platforms has changed the paradigm. Studies recruiting health-care personnel have often been a target for such research studies with mixed responsiveness [4]. Varied trials evaluating the impact of incentives on survey responsiveness have exclusively focused on European and North American respondents with a wide range of results [5–7].

Published research has been growing at an estimated annual global rate of 9–11% per year, with studies from non-western countries likely driving this growth [8, 9]. Countries such as China, India, Singapore and South Korea devote relatively large percentages of their gross domestic product to funding research endeavors or higher education [10]. Despite the contributions of the international community to the worldwide body of knowledge, data regarding international response rates for surveys remains scarce. The methodologies that produce reasonable response rates in Europe and United States may not be as effective in a broader, international cohort due to distinct perceptions and motivations in behavior that are culturally specific [11, 12]. Such prior efforts to motivate response in western countries include: offering a cup of coffee or small monetary incentives of $0–10 [7, 11, 12].

The importance of cultural variables in this context is unknown. From a neurocognitive perspective, some motivation may be inherent such as personal gain or participation in altruism [13]. To that end, we created a nested randomized control trial (RCT) within a prospective cohort study to assess the impact of incentives on survey responsiveness in a global audience of biomedical researchers. We hypothesized that altruism (via a donation to an international charity) as well as personal financial gain would improve international survey responsiveness, despite cultural differences.

## Main text

### Methods

The RCT was nested within a cohort study investigating open access journal publishing practices, as such it qualified as a Studies Within A Trial (SWAT) [14]. As part of the original study, we identified a cohort of biomedical researchers with available contact information, baseline English fluency, internet access, and assumption of professional degrees (given publication of scholarly work). The nested RCT study population was generated by randomly selecting editors and authors from a list of open access biomedical journals with predominantly online publishers.

**Fig. 1:**
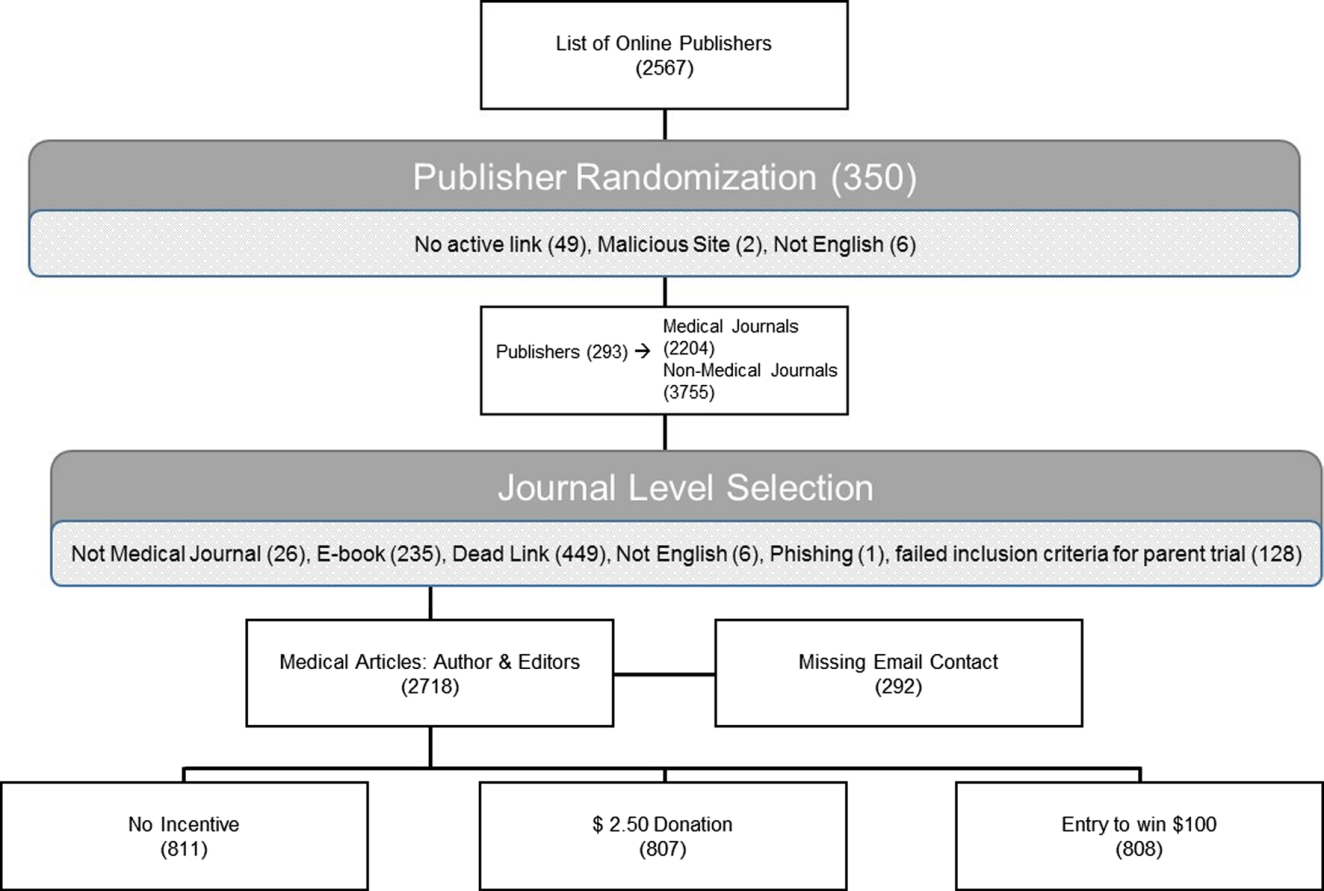
CONSORT flow diagram for included cohort

The parent study sought to understand author and editor attitudes regarding predatory publishing. Using a well-known list of potential predatory journals we randomly selected 350 publishers and their associated 2204 biomedical journals [15]. Journals were cross referenced with the Directory of Open Access, Open Access Scholarly Publishers Association and U.S. National Library of Medicine to eliminate mainstream open access publishers [16–18] 1359 biomedical journal articles ultimately met inclusion criteria. Authors’ journals met Medline criteria for biochemical research: “[Journals] predominantly devoted to reporting original investigations in the biomedical and health sciences, including research in the basic sciences; clinical trials of therapeutic agents; effectiveness of diagnostic or therapeutic techniques; or studies relating to the behavioral, epidemiological, or educational aspects of medicine” [19]. A summary of the trial structure can be found in Fig. 1. Subjects without publicly available contact information and non-English language journals were excluded. Once journals were identified, a single article from each journal was selected. The corresponding author and editor email addresses, year of publication (in the case of articles), and country of origin were recorded for that article.

A priori we planned for three groups: no incentive (control), altruistic donation, and financial incentive. Sample size calculations dictated 686 potential participants per group were required to detect an effect of a 5% difference in response rate with a Type I error rate of 5% and 80% power. Ultimately, we obtained 2426 email addresses allowing for 808 per group with the expectation that 7.5% of emails addresses would be inactive. Post-hoc power calculations based on the final number of working email addresses suggested we were powered to detect a 4.8% difference between groups.

The groups were designed as follows: (A) no incentive for participation to serve as the control group, (B) a $100 (or local currency equivalent) gift card for participation, and (C) a $2.50 altruistic donation to rotary club (https://www.rotary.org/) on behalf of the participant. Rotary International is a non-denominational international charity with 35,000 worldwide clubs that have been instrumental in multiple projects including the fight to eradicate Polio. All subjects were randomized using a random number generator in Stata (CollegeStation, Texas) and their corresponding email addresses were distributed into each group (Fig. 1).

An automated survey invitation was sent to each email stating the incentive, as such participants were not blind. However, subjects were unaware there were different incentives for other invitees. Each email invitation was personalized with the individual’s name in an automated fashion to increase the likelihood of individuals reading the email and completing the survey. Authors were blinded to group assignments while surveys were administered. A response was defined as complete or incomplete. Data analysis took place in a blinded fashion based on three groups of unknown incentives. Once data collection and data analysis was complete, authors were unblinded to allow for distribution of incentives and final formulation of the manuscript. All data was anonymized, and stored in REDCap with 1 automated reminder sent to non-responders 10 days after the initial survey was sent. Study data were collected and managed using REDCap electronic data capture tools hosted at UCSF [20].

Summary statistics were used to describe the cohort. Means and standard deviations were used for continuous variables. Frequency tables were used for categorical variables. Chi squared statistic was used to compare frequencies between groups. Developed nation status was based on World Bank listing for high-income countries [21]. Statistics were calculated using Stata 15. Institutional Review Board of the University of California-San Francisco reviewed and approved the study (IRB # 18-25351).

### Results

**Fig. 2:**
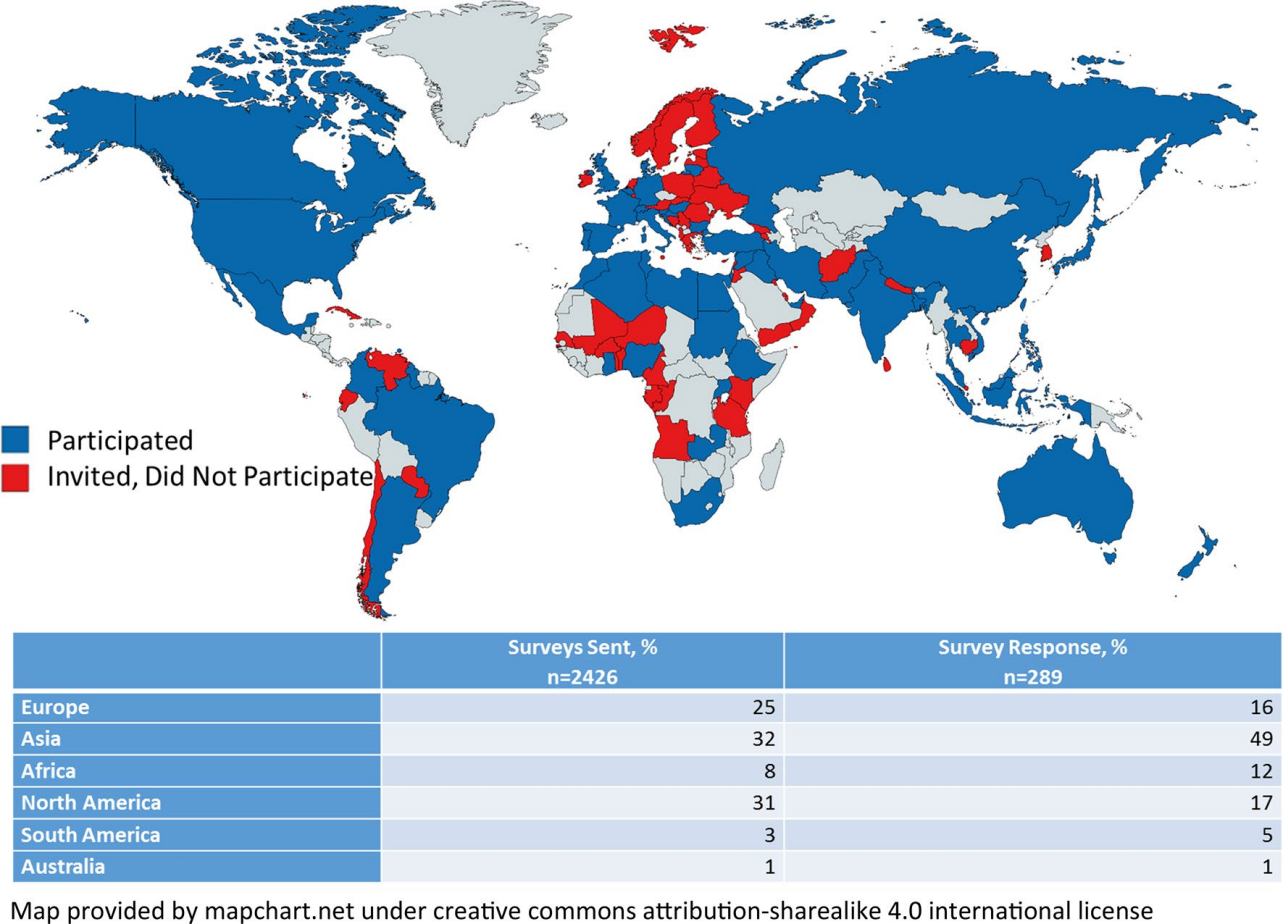
Distribution of global survey responses

Overall, 2426 email addresses from 111 countries were collected. 199 (8%) of email contact information resulted in a return to sender response, leaving a final 2227 perspective participants. After the initial survey was disseminated, 7.6% of individuals responded to the survey invitation and this increased to 13.0% after reminders were sent. The majority of potential respondents had published research within the last 2 years: 59.7% in 2018 and 17.7% from 2017. Response rates amongst the groups were: 13.3% for the control group, 14.4% for the gift card group, and 11.1% for the altruistic group (p = 0.17) (Table 1). When examining country of origin, India, the United States, and China were most represented but global participation was noted (Fig. 2). Based on unsolicited email replies to the survey invitation from potential respondents, 38 (1.7%) participants could not access the survey electronically from Nigeria or Cuba. It is unknown if similar difficulties limited responses in other countries.

**Table 1:** Response rates by incentive group

	Total invited	Faulty email	Final sent	Surveys responses n, %
		n, %	n, %	
Control (A)	811	54 (6.7)	757 (93.3)	101 (13.3)
Incentive (B)	807	72 (8.9)	735 (91.1)	106 (14.4)
Incentive (C)	808	73 (9.0)	735 (91.0)	82 (11.1)

The differences in survey response for those offered a gift card for participation vs. control 1.1% (*p *= 0.55) nor those participating in donation vs. control 2.2% (*p *= 0.20) were significant. Likewise, there were no differences in the response rate by country (p = 0.49). The year of publication for the articles from which emails were derived also did not impact response rate (p = 0.52).

The parent study was designed to assess differences between authors and editors; hence subgroup analysis of response by occupation was performed. Editors accounted for 47.7% of invited survey participants. Editor response rates was significantly lower than those for authors (6.5% vs. 18.9%; p-value < 0.01). There was a lower response rate among authors and editors from high-income countries; accounting for 35% and 42% of the cohort respectively. There was no significant difference in responses based on incentive group among authors (p = 0.55) and editors (p = 0.36).

### Discussion

To our knowledge, this is the first study to assess a donation on behalf of the participant to motivate global survey response. Given increased level of education may be correlated with greater altruism and altruism may be a universal motivator, we hypothesized international biomedical researchers and editors with high levels of education would be particularly sensitive to this approach [22]. Ultimately, the single most influential factor in our study was not altruism, but a reminder email, which increased our response by 41%. Overall, our response rate was 13% with no measurable impact from our intervention [12]. Previous work using altruism as motivation is rare: a donation to a charity germane to nephrologists’ clinical practice did not alter survey response in a small trial in Canada [23].

Prior work has been mixed as to whether financial motivations impact survey response and what monetary amount is most effective. The financial incentive equivalent to a cup of coffee, did not influence survey response rates in a trial of 472 patients [7]. Given the wide range in global coffee consumption and eating habits, specific food or beverage incentives are unlikely to strike a chord globally [24]. In other work, incentive amounts ranging from 0 to $10, did not dramatically alter survey responsiveness among physicians. On the other hand, 66 subject matter experts agreed small financial incentives may improve survey responses in randomized control trials [25]. Interestingly, the timing of incentive delivery may be important, a pre-survey $50 dollar incentive improved physician survey response in recent work [26]. A review by Pit et al. recently suggested monetary and nonmonetary incentives were more effective than no incentive (with upfront large monetary incentives most effective) [4]. Despite our digital age, postal surveys also fare better [4]. Given a lack of high quality randomized data, it is hard to definitively conclude if incentives are better than no incentives; hence the motivation for our study.

Our target audience was highly global with potential responders in 111 countries adding complexity to at first glance a simple survey request. Local culture, currency, and language may impact study participant behavior in unforeseen ways [27]. While translating surveys into a local dialect or using a single language are options for the researcher, survey adaptation or cross-cultural validation may be necessary for scientific vigor and to improve responses [28, 29]. Survey respondents from certain countries could not even access our electronic survey. Which countries were affected remained unknown, but an email confirmed at least respondents in Nigeria and Cuba could not access the survey link perhaps due to internet censorship in those locations [30]. Novel survey distribution via smart devices [31] or gamification of surveys may provide innovative methods to improve survey responses [32] but research is lacking on these novel methods. In particular how these methods will be viewed through a cross-cultural lens is largely unknown.

### Conclusion

Survey research emanating from the United States targeting global participation incurs unique cultural, technological, and financial challenges. Altruistic donation nor personal financial gain seem to motivate a global audience of biomedical researchers to respond. The best strategy to optimize global participation in electronic survey remains unknown. Until a universal incentive is discovered, cross-cultural validation and locality-specific incentives should be considered in future trials to improve responses in global research.

## Limitations

While potential targets had published a manuscript in English, this may not have been their first language and confusion regarding our study may have reduced participation. In selecting our charity, the authors did our best to select one with global, non-political focus; nonetheless, the charity may not be familiar or a worthy cause to all potential participants. Our donation and monetary reimbursement amounts were limited by budget, but certainly different or increased amounts may motivate individuals differently. Incentives were described in US dollars, given exchange rates, these amounts could be interpreted quite differently depending on the respondents’ country of origin. We suspect that these limitations would be distributed non-differentially across our randomized groups and therefore not ultimately effect the results. Our potential respondents were quite heterogeneous in terms of research and career focus, internet access, and country. Given limited data regarding non-respondents, we were unable to perform valid logistic regression to assess predictors of positive survey response. Overall low survey response may be explained by survey fatigue, lack of interest in topic, or characterization of our email invitation as spam. The parent trial sought authors and editors involved with predatory publishing, who may not be forthcoming in participating in survey research.
